# Activated Cdc42-associated kinase 1 (ACK1) binds the sterile α motif (SAM) domain of the adaptor SLP-76 and phosphorylates proximal tyrosines

**DOI:** 10.1074/jbc.M116.759555

**Published:** 2017-02-10

**Authors:** Youg R. Thaker, Asha Recino, Monika Raab, Asma Jabeen, Maja Wallberg, Nelson Fernandez, Christopher E. Rudd

**Affiliations:** From the ‡ Cell Signaling Section, Department of Pathology, University of Cambridge, Tennis Court Road, Cambridge CB2 1QP, United Kingdom,; the § Department of Obstetrics and Gynecology, School of Medicine, J. W. Goethe University, Theodor-Stern-Kai 7, 60590 Frankfurt, Germany,; the ¶ School of Biological Science, University of Essex, Wivenhoe Park, Colchester CO4 3SQ, United Kingdom,; the ‖ Division of Immunology-Oncology Research Center Maisonneuve-Rosemont Hospital, Montreal, Quebec H1T 2M4, Canada, and; the ** Département de Medicine, Université de Montréal, Montreal, Quebec H3C 3J7, Canada

**Keywords:** cell signaling, cluster of differentiation 4 (CD4), immunology, non-receptor tyrosine kinase (nRTK), protein phosphorylation, sterile α motif (SAM), T cell

## Abstract

The adaptor protein Src homology 2 domain-containing leukocyte phosphoprotein of 76 kDa (SLP-76) plays a crucial role in T cell activation by linking antigen receptor (T cell receptor, TCR) signals to downstream pathways. At its N terminus, SLP-76 has three key tyrosines (Tyr-113, Tyr-128, and Tyr-145, “3Y”) as well as a sterile α motif (SAM) domain whose function is unclear. We showed previously that the SAM domain has two binding regions that mediate dimer and oligomer formation. In this study, we have identified SAM domain-carrying non-receptor tyrosine kinase, activated Cdc42-associated tyrosine kinase 1 (ACK1; also known as Tnk2, tyrosine kinase non-receptor 2) as a novel binding partner of SLP-76. Co-precipitation, laser-scanning confocal microscopy, and *in situ* proximity analysis confirmed the binding of ACK1 to SLP-76. Further, the interaction was induced in response to the anti-TCR ligation and abrogated by the deletion of SLP-76 SAM domain (ΔSAM) or mutation of Tyr-113, Tyr-128, and Tyr-145 to phenylalanine (3Y3F). ACK1 induced phosphorylation of the SLP-76 N-terminal tyrosines (3Y) dependent on the SAM domain. Further, ACK1 promoted calcium flux and NFAT-AP1 promoter activity and decreased the motility of murine CD4^+^ primary T cells on ICAM-1-coated plates, an event reversed by a small molecule inhibitor of ACK1 (AIM-100). These findings identify ACK1 as a novel SLP-76-associated protein-tyrosine kinase that modulates early activation events in T cells.

## Introduction

T cell receptor (TCR)^2^ signaling is mediated by the activation of protein-tyrosine kinases such as p56^lck^ and ZAP-70 (1, 2). In mature T cells, CD4 and CD8 molecules bind to p56^lck^ (3–5), which then phosphorylates the intracellular immune receptor tyrosine-based activation motifs in the TCR-associated CD3 subunits for ZAP-70 recruitment. Binding of ZAP-70 to CD3 ζ is an initial step in the transduction of signaling cascades, resulting in the phosphorylation of various molecules, including the linker for the activation of T cells (LAT) and the SH2 domain-containing leukocyte protein of 76 kDa (SLP-76) (1, 2, 4, 6–9). The phosphorylated LAT and SLP-76 proteins function as scaffolds to recruit many other signaling molecules to form multimeric complexes (10, 11). SLP-76 has been shown to be needed for thymic differentiation and mature T cell function (12, 13). Its loss impairs the activation of phospholipase Cγ1 (PLCγ1) calcium mobilization, and adhesion (14–16). Upon TCR engagement, SLP-76 migrates to the immunological synapse (17) and forms microclusters (18). Adaptor LAT in motile vesicles can be also found with surface SLP-76 microclusters during T cell-APC interaction (19). SLP-76 can also back-regulate ZAP-70 microcluster formation, an event that could ensure that signaling components are in balance for optimal T cell activation (20). In addition, SLP-76 can bind to RanGAP1 of the nuclear pore complex and directly regulate the nuclear transport of the transcription factors NFATc1 and NF-κB (21).

Structurally, SLP-76 is comprised of four domains: a sterile α motif (SAM) domain, an acidic region with three tyrosine residues (Tyr-113, Tyr-128, and Tyr-145) at the N terminus, a central proline-rich domain, and a C-terminal Src homology 2 (SH2) domain. The N-terminal SAM domain is essential for optimal thymic differentiation and cell activation (22, 23). SAM domains are known to interact with themselves or with other SAM domain-containing proteins or non-SAM domain-containing proteins in a homotypic or heterotypic fashion (24). This binding versatility allows them to regulate diverse biological functions ranging from signal transduction to transcriptional regulation (25). Despite this, the molecular events regulating SAM domain-dependent functions of SLP-76 have yet to be deciphered.

The N terminus tyrosines (3Y) are essential for supporting T cell effector functions, including NFAT promotor activity (20, 21, 26). ZAP-70-mediated phosphorylation of the SLP-76 tyrosine residues 113, 128, and 145 constitutes a key TCR signaling event (14, 27). Cells lacking ZAP-70 have diminished phosphorylation of SLP-76 (28). Genetic deletion of ZAP-70 results in severe combined immunodeficiency in both mouse and human, with a dramatic reduction in the number of peripheral CD4 and CD8 T cells, making it difficult to assess the role of additional kinases in peripheral signaling (29). On the other hand, interleukin 2 tyrosine kinase-deficient cells with defective phosphorylation of PLCγ1 and calcium mobilization (30) can be complemented with a related kinase, resting lymphocyte kinase, which phosphorylates SLP-76, leading to the phosphorylation of PLCγ1, activation of ERKs, and the synergistic up-regulation of TcR-driven IL-2 NFAT/AP-1 transcription (31).

Recently, we have identified a unique role for the SAM domain of SLP-76 in the regulation of T cell activation (22). We observed that the SAM domain is comprised of two binding regions that lead to the formation of dimers and higher-order oligomers. SAM domain (1–78 residues)-deficient SLP-76 (ΔSAM) fails to form microclusters and to enhance anti-CD3-driven NFAT/AP-1 transcription. Mapping revealed an α helix 5 (H5) within the SAM domain that contributed to its ability to self-associate. Retention of H5 in the absence of other helices (H1-H4) was sufficient to support T cell activation and microcluster formation, albeit at a higher rate compared with wild-type SLP-76 (22).

Despite this progress, the mechanisms underlying SAM domain functions remain unclear. In this study, we have examined the capacity of the SLP-76 SAM domain to interact with other SAM domain-carrying proteins. We investigated kinases retrieved from the Sugen/Salk Kinase Database (KinBase) present in the human kinome (32, 33) that contained several SAM kinases with N-terminal SAM domains. We reasoned that the N-terminal domains may have evolved an ability to interact with other N-terminal domains. This led to the identification of activated cell division cycle 42 (Cdc42)-associated tyrosine kinase 1 (ACK1) as a kinase that associates with SLP-76 in a SAM-dependent manner and regulates its phosphorylation and aspects of T cell signaling.

## Results

### ACK1 binds SLP-76 via SAM-SAM interaction

**Figure 1.**
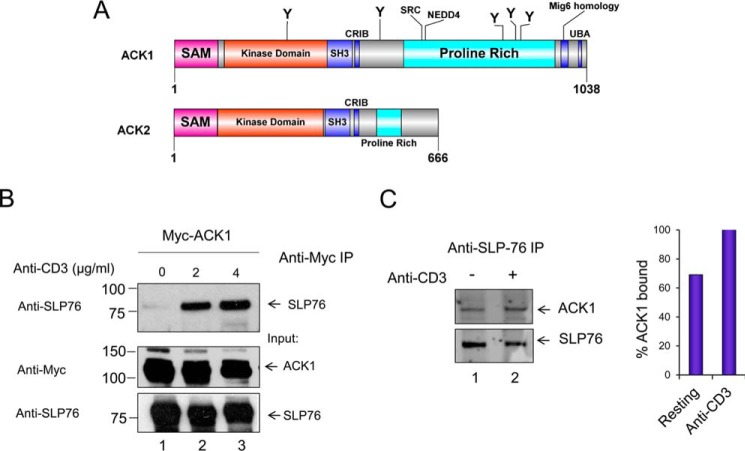
**SLP-76 binds to ACK1.** *A*, representation of the ACK1 and ACK2 domain architecture. *UBA*, ubiquitin-associated; *CRIB*, Cdc42- and Rac-interactive binding motif. *B*, anti-SLP-76 co-precipitates ACK1. Jurkat cells were transfected with Myc-tagged ACK1, left resting (*lane 1*), or stimulated at 24 h with soluble anti-CD3 (2 μg/ml, *lane 2*) or (4 μg/ml, *lane 3*) for 5 min, followed by immunoprecipitation (*IP*) with anti-Myc and blotting with anti-Myc or SLP-76. *C*, anti-SLP-76 co-precipitates ACK1 from primary mouse T cells. Immunoprecipitation used anti-SLP-76 from primary T cells isolated from mouse spleen. Primary T cells were either left resting (*lane 1*) or incubated for 5 min with anti-CD3 (*lane 2*).

The N terminus SAM domain of SLP-76 is essential for optimal T cell signaling, microcluster formation, IL-2 production, and NFAT activity (22). However, little is understood regarding molecular events regulating these functions. Previous studies have documented the importance of ZAP-70-mediated phosphorylation of SLP-76 in the regulation of T cell responses (28). It has remained an open question whether other kinases exist in T cells that mediate SLP-76 phosphorylation and promote its function. In particular, we were interested in whether SAM domain-carrying kinases could bind to SLP-76, leading to its phosphorylation. A search of several databases, including KinBase, led to the identification of the N-terminal SAM domain kinases ACK1 (or Tnk2, tyrosine kinase non-receptor 2) and ACK2 (or Tnk1, tyrosine kinase non-receptor 1) (Fig. 1*A*). Intriguingly, the highest expression levels of ACK1 are found in the spleen, the thymus, and the brain (34). Analysis of entries in the microarray database also showed the highest expression of ACK1 in lymphocytes, such as CD4 conventional and regulatory T cell populations, which was further confirmed by quantitative real-time-PCR and Western blotting (supplemental Fig. S1).

**Figure 2.**
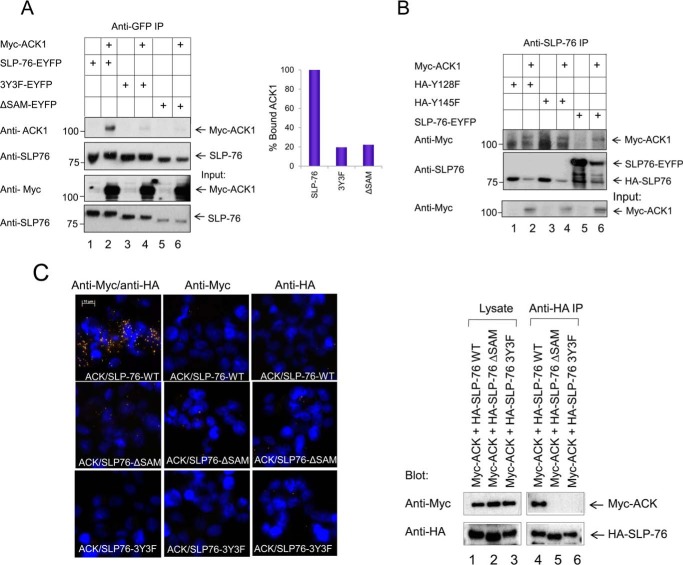
**ACK1 binding to SLP-76 is dependent on the SLP-76 SAM domain and three proximal tyrosine residues: Tyr-113, Tyr-128, and Tyr-145.** *A*, ACK1 binding to the SLP-76 mutant in HEK293T cells. Various SLP-76 mutants (wild-type SLP-76, proximal tyrosine mutant (3Y3F), or SAM-deficient (ΔSAM) SLP-76) were expressed with Myc-tagged ACK1. Cells were transfected for 24 h before harvesting. Lysates were subjected to immunoprecipitation (*IP*) with anti-GFP antibody, capturing SLP-76 and bound ACK1 (*lane 2*). No binding was seen with ΔSAM (*lane 6*) or the 3Y3F mutant (*lane 4*). The percentage of bound ACK1 is shown in *the right panel. B*, unlike the 3Y3F mutant, the HA-tagged single tyrosine mutants Y128F and Y145F failed to disrupt the SLP76-ACK1 complex (*lanes 2* and *4*). *C*, *left panel*, *in situ* proximity ligation assay (PLA) showing co-localization of Myc-ACK1 with HA-SLP-76 (*red dots*, *top row*) but not with SLP76-ΔSAM (*center row*) or 3Y3F mutants (*bottom row*). No binding was seen in controls. *Right panel*, binding assessed by immunoprecipitation from the lysates of samples used in the PLA. The results in *C* are representative of two experiments and in *A* and *B* representative of four experiments performed in two different laboratories.

SAM-containing proteins can bind other proteins and form homo- or heterodimers and may even form multimers (22, 35). To evaluate the possible interaction of ACK1 with endogenous SLP-76, Myc-tagged ACK1 was expressed in Jurkat T cells by transfection, followed by ligation with 2 or 4 μg/ml of soluble anti-CD3 for 5 min. ACK1 protein was precipitated with anti-Myc antibody (Fig. 1*B*). A small amount of endogenous SLP-76 was co-precipitated with anti-Myc (Fig. 1*B*, *lane 1*), which was markedly increased with anti-CD3 (Fig. 1*B*, *lanes 2* and *3*). As controls, anti-Myc detected Myc-ACK1 and anti-SLP-76 detected SLP-76 in cell lysates (Fig. 1*B*, *bottom panels*). These results indicated that endogenous SLP-76 could be co-precipitated with transfected ACK1, and the association was increased with anti-CD3 stimulation. We also observed binding in murine primary resting mouse T cells by precipitation using an anti-SLP-76 antibody followed by anti-ACK1 blotting (Fig. 1*C*). Purified CD4^+^ T cells were isolated from mouse spleen and activated with soluble anti-CD3 for 5 min. Anti-SLP-76 co-precipitated ACK1 in resting cells (Fig. 1*C*, *left panel*, *lane 1*) that was increased with anti-CD3 (Fig. 1*C*, *left panel*, *lane 2*, and *right panel*). Binding between SLP-76-EYFP and Myc-ACK1 could also be observed in transfected non-hematopoietic HEK293T cells (Fig. 2, *A*, *lane 2*, and *B*, *lane 6).* Additionally, *in situ* proximity hybridization (PLA) of ACK1 and SLP-76 gave a positive signal that was indicative of close proximity in HEK293T cells (Fig. 2*C*, *top left panel*, *yellow dots*). Together, these findings indicate, for the first time, that ACK1 and SLP-76 bind to each other in different cell lines, including Jurkat and primary T cells.

To assess the binding sites between ACK1 and SLP-76, we expressed various SLP-76 mutants in non-hematopoietic HEK293T cells with Myc-tagged ACK1 (Fig. 2*A*). Anti-SLP-76 co-precipitated the Myc-ACK1 protein and wild-type SLP-76 (Fig. 2*A*, *lane 2*). By contrast, anti-SLP-76 co-precipitated a much reduced ACK1 band from cells expressing the mutant SLP76-ΔSAM (residues 1–78 deletion) (Fig. 2*A*, *lane 6*) or SLP-76 lacking proximal tyrosines 3Y3F (tyrosine-to-phenylalanine mutants) (Fig. 2*A*, *lane 4*). By contrast, in another experiment, a single mutation of Tyr-128 or Tyr-145 to phenylalanine did not disrupt binding (Fig. 2*B*, *lanes 2* and *4*), suggesting possible cooperativity among the three tyrosine residues.

Further, in an *in situ* proximity ligation assay (PLA), anti-Myc and anti-HA antibodies were employed with the Duolink^TM^ detection system in HEK293T cells (Fig. 2*C*). Cells were transfected with Myc-ACK1 to bind anti-Myc and HA-tagged SLP-76 to bind anti-HA. From this, a strong signal was seen between ACK1 and SLP-76 (Fig. 2*C*, *left panel*, *top left*). This was consistent with the co-precipitation of Myc-ACK1 in anti-HA precipitates of HA-SLP-76 (Fig. 2*C*, *right panel*, *lane 4*). By contrast, co-expression of Myc-ACK1 with HA-SLP76-ΔSAM (Fig. 2*C*, *left panel*, *center row*) or HA-SLP76–3Y3F (Fig. 2*C*, *left panel*, *bottom row*) failed to show a positive PLA signal or to be co-precipitated in anti-HA SLP-76 precipitations (Fig. 2*C*, *right panel*, *lanes 5* and *6*). Collectively, these findings showed that the interaction between ACK1 and SLP-76 was dependent on the SLP-76 SAM domain and proximal tyrosine residues at 113, 128, and 145.

**Figure 3.**
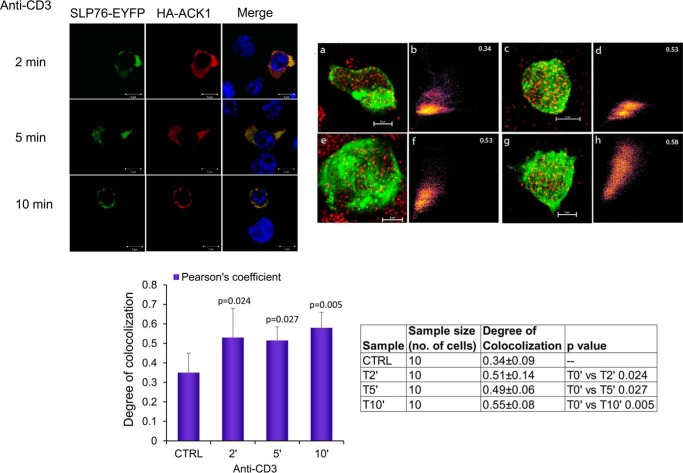
**Laser confocal imaging shows that ACK1 co-localizes with SLP-76 in Jurkat T-cells.** *Left panel*, laser confocal images showing co-localization of SLP-76 (*green*) with ACK1 (*red*), with nuclear DAPI shown in *blue*. Cells were treated with anti-CD3 for 2 min (*top row*) to 10 min (*bottom row*). *Top right panel*, 3D projections of deconvolved confocal images of SLP-76 (*green*) and ACK1 (*red*) in T cells. *a*, *c*, *e*, and *g*, confocal microscope images of Jurkat T cells treated with anti-CD3 for 0, 2, 5, and 10 min, respectively. Each image is an overlay of green (SLP-76-EYFP) and red (HA-ACK1). *b*, *d*, *f*, and *h*, 2D co-localization histograms at 0, 2, 5, and 10 min, respectively. Pearson's correlation coefficient value is shown in the *top right corners. Bottom right panel*, mean degree of colocalization ± S.D. Data were statistically analyzed using one-way analysis of variance. The *p* values for each treated group represent statistically significant differences compared with the control group (*bottom right panel*, *table*). All treated groups show a significant difference compared with the control resting group (0 min), whereas 10 min appears to be most significant (*p* = 0.005) among all groups. Images are representative of three independent experiments performed in two different laboratories. *CTRL*, control.

Consistent with these findings, we next showed the co-localization of SLP-76 and ACK1 using laser-scanning confocal microscopy of Jurkat cells on anti-CD3-coated glass coverslips (Fig. 3, *left panel*). Cells were imaged over a time course in response to anti-CD3 ligation. Representative 3D projections of deconvolved images of T cells treated with anti-CD3 (Fig. 3, *top right panel*, *a* (0 min), *c* (2 min), *e* (5 min), and *g* (10 min)) were used to assess the co-localization coefficient (Fig. 3, *top right panel*, *b*, *d*, *f*, and *h*). An increase in co-localization was seen in response to anti-CD3 by 2 min, and this remained stable until 10 min. Pearson's correlation coefficient values of 0.51 at 2 min and 0.49 at 5 min revealed significant overlap between SLP-76 (*green*) and ACK1 (*red*) upon stimulation compared with the resting control group (Fig. 3, *bottom panel*, *histogram*). Antibody specificity controls are shown in supplemental Fig. S1*D*). These findings provide strong evidence of SLP-76 binding to ACK1.

### ACK1 phosphorylates SLP-76-proximal tyrosine residues

**Figure 4.**
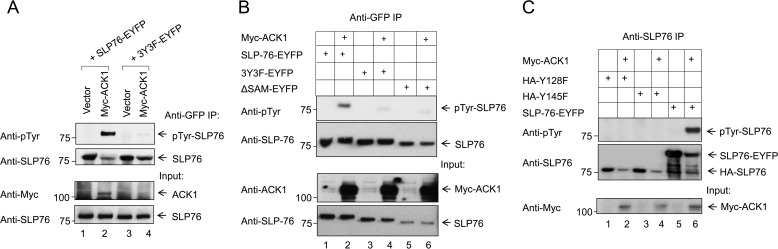
**ACK1 mediates phosphorylation of SLP-76 3Y proximal tyrosines.** *A*, phosphorylation of SLP-76 by ACK1 was assessed by co-transfecting HEK293T cells with ACK1 and WT SLP76-EYFP (*lane 2*) or tyrosine mutant SLP-76 (*3Y3F-EYFP*, *lane 4*) for 24 h, followed by SLP-76 immunoprecipitation (*IP*) with anti-GFP, and total tyrosine phosphorylation was assessed by 4G10 (anti-Tyr(P)) antibody (*first blot*). SLP-76 immunoprecipitation efficiency was assessed by anti-SLP76 blotting (*second blot*), whereas transfection efficiency was assessed by blotting of lysates with anti-Myc (for ACK1) and anti-SLP-76 (for WT and 3Y3F SLP-76). *B*, ACK1-mediated phosphorylation requires SLP-76 to possess an intact N terminus, as shown by loss of phosphorylation in SLP76-ΔSAM (*lane 6*) and 3Y3F (*lane 4*) SLP-76 mutants. WT SLP-76 (*lane 2*) was efficiently tyrosine-phosphorylated. No detectable phosphorylation was seen in control cells co-transfected with empty Myc-tagged vector (*lanes 1*, *3*, and *5*). *C*, point mutation of Tyr-128 or Tyr-145 abrogated tyrosine phosphorylation of unmutated Tyr-113 and Tyr-145 (*lane 2*) or Tyr-113 and 128 (*lane 4*), respectively. Co-transfection of SLP-76 or its mutants with empty vector (*lanes 1*, *3*, and *5*) served as a negative control. All blots shown are representative of at least three to four independent experiments. For comparison purposes, the data for Fig. 4, *B* and *C*, are derived using the same input lysates as used in Fig. 2, *A* and *B*, respectively.

*In vitro* and *in vivo* studies have demonstrated that tyrosines 113, 128, and 145 in the acidic N-terminal region of SLP-76 are critical for supporting T cell functions (27, 28). These tyrosines are phosphorylated by ZAP-70 kinase (28, 36). Given our evidence that SLP-76 binds to ACK1, we next investigated whether ACK1 can also phosphorylate SLP-76. We co-expressed SLP-76-EYFP or the 3Y3F-SLP76-EYFP mutant with ACK1 or empty vector in HEK293T cells, followed by precipitation with anti-GFP and blotting with various antibodies (Fig. 4). Expression of SLP-76 with empty vector revealed no detectable tyrosine phosphorylation (Fig. 4*A*, *lane 1*). By contrast, ACK1 co-expression with SLP-76 resulted in its significant phosphorylation (Fig. 4*A*, *lane 2*). Further, ACK1 failed to phosphorylate the SLP-76 tyrosine mutant 3Y3F lacking all three proximal tyrosines (Fig. 4*A*, *lane 4*).

In view of the result showing that deletion of the SAM domain disrupted binding of ACK1 to SLP-76 (Fig. 2*A*), we also assessed whether the loss of this domain affected tyrosine phosphorylation. SLP-76 wild-type, SLP76-ΔSAM, or 3Y3F were co-expressed with ACK1 (Fig. 4*B*). SLP-76 was then immunoprecipitated, and tyrosine phosphorylation was assessed by blotting. As before, wild-type SLP-76 became phosphorylated when co-expressed with ACK1 (Fig. 4*B*, *lane 2*). However, the SLP76-ΔSAM mutant (Fig. 4*B*, *lane 6*) and the 3Y3F mutant (Fig. 4*B*, *lane 4*) failed to become tyrosine-phosphorylated. The binding data showed that ACK1 sustained binding to Y128F and Y145F mutants, suggesting that two of three unmutated tyrosines are sufficient for binding (Fig. 2*B*). Based on this, we speculated that mutation of a single tyrosine residue in the 3Y motif will still permit phosphorylation of the remaining intact tyrosines (*i.e.* Tyr-113 and Tyr-145 when Tyr-128 is mutated and Tyr-113 and Tyr-128 when Tyr-145 is mutated). Unexpectedly, however, a point mutation of Tyr-128 or Tyr-145 to phenylalanine abolished phosphorylation of the entire 3Y motif (Fig. 4*C*). This finding demonstrates that ACK1 concurrently phosphorylates all three residues and requires an intact N-terminal SAM and the three N-terminal tyrosines. The Tyr-113 mutant was not included in this study. Therefore, the likelihood of Tyr-128 and Tyr-145 being co-phosphorylated in the absence of Tyr-113 could not be ruled out. However, these results suggest concurrent phosphorylation of tyrosines 113, 128, and 145.

### ACK1 increases calcium flux and NFAT transcription

**Figure 5.**
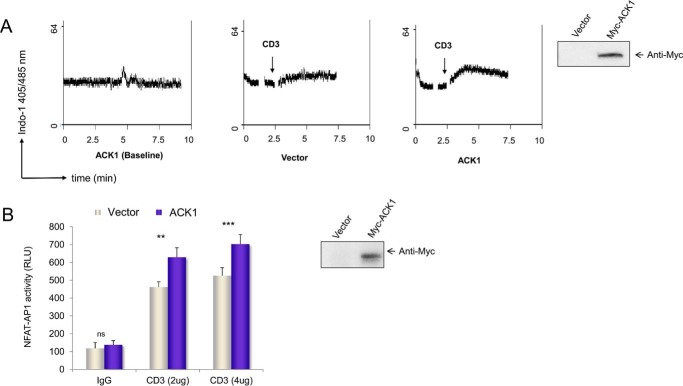
**ACK1 enhanced anti-CD3-induced calcium flux and NFAT activity.** *A*, primary CD4^+^ T-cells transfected with ACK1 (*first* and *third panel*s) or vector (*second panel*) were loaded with Indo-1 dye, followed by stimulation with anti-CD3 (0. 25 μg/ml). The plots show the ratio of bound to free dye on the *y* axis with time (on the *x* axis, in minutes). Calcium flux in response to anti-CD3 in vector-transfected (*second panel*) or ACK1-transfected (*third panel*) primary CD4^+^ T-cells is shown. The *first panel* shows the baseline without anti-CD3 stimulation. ACK1 expression was assessed by Western blotting (*fourth panel*). *B*, ACK1 results in enhanced NFAT-AP1 activity in Jurkat T cells transfected with exogenous Myc-ACK1 (*blue columns*) or vector (*silver columns*). Transfected cells were stimulated for 6 h with soluble anti-CD3 (2 μg/ml OKT3) and 1 μg/ml of rabbit anti-mouse IgG crosslinking antibody. 3×NFAT/AP1 promoter-driven luciferase activity was measured with the Dual-Luciferase assay kit (Promega). The data shown are normalized to *Renilla* luciferase and representative of at least two independent experiments. *ns*, not significant; **, *p* ≤ 0.01; ***, *p* ≤ 0.001); unpaired Student's *t* test (mean ± S.E.).

Emphasizing the critical role of the 3Y residues, previous studies have established that, in SLP-76-deficient cells, the 3Y3F mutant fails to rescue PLCγ1 phosphorylation, calcium flux, and NFAT transcriptional activity (37). To determine the functional consequence of ACK1 in this context, we made use of CD4^+^ primary mouse T cells transfected with ACK1 or a control vector (Fig. 5*A*). Cells were loaded with the intracellular calcium indicator Indo-1 dye and assessed for their ability to support the influx of extracellular calcium in response to suboptimal anti-CD3 concentrations. CD4^+^ T cells expressing ACK1 showed an enhanced influx of calcium in response to anti-CD3. Anti-Myc blotting confirmed the expression of transfected protein (Fig. 5*A*, *fourth panel*). Consistent with this, ACK1 exogenous expression also increased NFAT/AP1 promoter activity (Fig. 5*B*).

**Figure 6.**
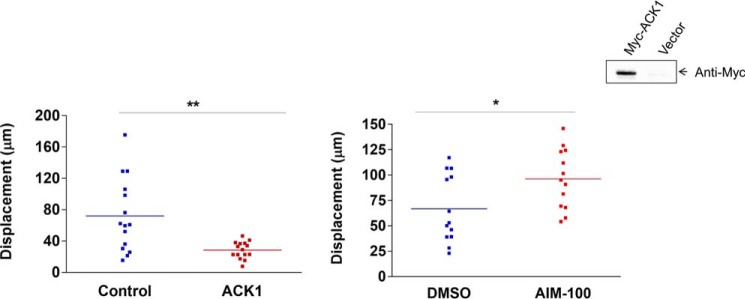
**ACK1 slows T cell motility.** *Left panel*, motility (displacement) of CD4^+^ T-cells on ICAM-1-Fc-coated plates that were pretransfected with either empty vector (*blue dots*) or ACK1 (*red dots*) measured by live-cell imaging. *Inset*, expression of ACK1 by Western blotting. *Right panel*, displacement of CD4^+^ cells that were pretreated with either ACK1 inhibitor (AIM-100) or control (DMSO), tracked on ICAM-1-Fc-coated plates. 15–20 individual cells (represented by *dots*) for each condition were analyzed by Volocity software. *, *p* ≤ 0.05; **, *p* ≤ 0.01; unpaired Student's *t* test (mean ± S.E.).

In addition, the effect of ACK1 on T cell motility was examined (Fig. 6). ACK1 has been implicated previously in hepatocellular carcinoma metastasis (38). We observed a decrease in the random motility of T cells upon exogenous ACK1 expression compared with wild-type cells on ICAM-1-coated plates (Fig. 6, *left panel*). Further, consistent with this, inhibition of endogenous ACK1 activity by pretreatment of cells with an ACK1 inhibitor (AIM-100) resulted in significantly increased cell motility compared with untreated cells. However, the detailed role of ACK1 in synapse formation and integrin signaling will be addressed in future studies. These results strengthen the evidence that the ACK1 gene is a positive regulator of T cell function and that deregulation of its function may lead to pathologies such as cancer (39).

## Discussion

The adaptor protein SLP-76 plays a pivotal role in the transmission of signals from the TCR to the transcriptional machinery (37). The identity of the full range of associated kinases that bind and phosphorylate SLP-76 is not known. Previous studies from us and others have shown that ZAP-70 phosphorylates SLP-76 in the modulation of its function (27, 28). Here we have identified a new non-receptor SAM domain-carrying protein-tyrosine kinase, ACK1, that binds to SLP-76, resulting in the phosphorylation of its key tyrosine residues at Tyr-113, Tyr-128, and Tyr-145. Binding was abrogated by the deletion of the SLP-76 SAM domain (ΔSAM) or by mutation of three key tyrosine (3Y3F) residues in the N terminus of SLP-76. Functionally, ACK1 promoted calcium flux and NFAT-AP1 promoter activity and decreased the random motility of murine CD4^+^ primary T cells on ICAM-1-coated plates. An increase in motility was observed upon ACK1 inhibition by the small molecule inhibitor AIM-100. These findings identify ACK1 as a novel SLP-76-associated protein-tyrosine kinase that phosphorylates SLP-76 in the modulation of early activation events in T cells.

We showed previously that the SAM domain of SLP-76 mediates adaptor oligomer formation and that its deletion causes loss of microcluster formation, NFAT transcription, and IL-2 production (22). To fully understand the function of the SAM domain, we hypothesized that it might also bind other kinases. In this context, given the importance of the SAM domain and the fact that SAM domains can bind to other SAM domains (35), we hypothesized that the domain might recruit a SAM domain-carrying kinase. Because no binding partner of the SLP-76 SAM domain was known, we mined the KinBase database, which identified two N-terminal SAM domain kinases, ACK1 and ACK2 (Fig. 1*A*). Quantitative real-time-PCR, Western blotting and microarray (Immgen), and RNA sequencing data^3^ confirmed ACK1 expression in T cells (supplemental Fig. S1). Interestingly, however, the related ACK2 was not expressed in T cells (supplemental Fig. S1) (40). ACK2, contrary to ACK1, lacks ubiquitin-associated (UBA) and mitogen-induced gene 6 (Mig-6) homology domains as well as most of the C terminus (41). However, both ACK1 and ACK2 are distinct in having an SH3 and Cdc42- and Rac-interactive binding motif (CRIB) domain not found in other non-receptor tyrosine kinases. Therefore, they are likely to differ in their mechanism of action. Homologous proteins are found in the mouse, cow, *Drosophila melanogaster*, and *Caenorhabditis elegans* (42, 43). Human and mouse ACK1 proteins are highly conserved (93.4% identity) (supplemental Fig. S1).

Our findings clearly showed that the loss of the SLP-76 SAM domain abrogated its ability to bind to ACK1. Interestingly, the mutation of the proximal 3Y tyrosines (Tyr-113, Tyr-128, and Tyr-145) also disrupted this interaction. Single mutation of either the Tyr-128 or Tyr-145 residue disrupted ACK1-SLP76 complex formation. Whether the proximity of the tyrosines to the SAM domain influences SAM function (*i.e.* alters the conformation) or whether they exert an effect via an aspect of direct recognition is not clear. In either case, these findings present ACK1 as a new binding partner of SLP-76 with clear evidence that this binding occurred via the N terminus of the SLP-76 SAM domain. The interaction may account in part for the importance of the SLP-76 SAM domain in mediating optimal T cell activation.

In this context, we also found that ACK1 has the capacity to specifically phosphorylate the SLP-76 Tyr-113, Tyr-128, and Tyr-145 residues, as shown by its failure to phosphorylate 3Y3F mutants. Previous studies by us and others identified ZAP-70 as the kinase responsible for SLP-76 phosphorylation (27, 28). Several studies have established a role of ACK1 as a major integrator of receptor signals in pathways like EGF receptor, IGF-1, and insulin (39). Whether ZAP-70 and ACK1 act independently or in synergy remains to be investigated. ACK1-mediated phosphorylation was dependent on its binding to SLP-76 and was abrogated by loss of the SLP-76 SAM domain. Contrarily, there is no evidence of ZAP-70 binding to SLP-76, making ACK1 a unique kinase (27). Because SAM-deficient ACK1 lacks kinase activity (34, 44), its phosphorylation could be a direct consequence of SAM-SAM interaction. This is the first reported occurrence of kinase activity mediated by SAM domain binding in T cells. The ACK1-SLP76 complex is therefore likely to operate in an autoregulatory manner, where SAM binding is needed to recruit ACK1, which, in turn, phosphorylates tyrosines. Loss of tyrosine phosphorylation of 3Y motifs upon single mutations (Y128F or Y145F) suggests cooperativity among tyrosines, as noted previously (45).

Further studies will be needed to assess whether ACK1 can also cooperate with the interleukin 2 tyrosine kinase pathway, where the kinase phosphorylates PLCγ1 for the regulation of calcium mobilization (30). ACK1 may also cooperate with resting lymphocyte kinase, which, as we showed previously, can also phosphorylate SLP-76 to enhance the activation of PLCγ1, ERK, and NFAT/AP-1 transcription (31).

Previous studies have shown that mutation of Tyr-113 and Tyr-128 of SLP-76 (the residues needed for binding to VAV1 and NCK (non-catalytic region of tyrosine kinase adaptor protein)) results in defective PLCγ1 phosphorylation, calcium flux, and NFAT activity (37). Because ACK1 directly phosphorylates 3Y, we assessed its influence on calcium flux in primary CD4^+^ cells. Under suboptimal anti-CD3 concentrations, exogenous ACK1 led to enhanced calcium flux. This result implies that ACK1-mediated tyrosine phosphorylation of SLP-76 influences signaling via the PLCγ1-calcium axis, which feeds into increased nuclear translocation of NFAT. This was confirmed by an increase in NFAT/AP1 luciferase promoter activity observed upon anti-CD3 stimulation in ACK1-transfected Jurkat T cells.

SLP-76 also has a well documented role in cytoskeleton reorganization and motility (46, 47). Our findings also indicated involvement of ACK1 in T cell motility. Motility requires alterations in the affinity of LFA-1 to its ligand ICAM-1 by the signaling events that induce the contractile forces needed for cell movement. Therefore, motility was measured as random movements on ICAM-1-coated plates. Overexpression of ACK1 in preactivated primary CD4^+^ T cells decreased the motility on ICAM-1, which was reversed by inhibiting ACK1 activity using a small molecule inhibitor, AIM-100. The interplay of the LFA-1/ACK1 axis with other integrin signaling proteins remains to be determined. Blocked motility by ACK1 could be linked to a reduced LFA-1 cluster or changes in ADAP (adhesion- and degranulation-promoting adapter protein) binding to SLP-76, involved in outside-in signaling (47). The detailed role of ACK1 in motility, polarization, and synapse formation needs to be address in future studies, but the data gathered in this study indicate that ACK1 could well play a critical role in T cell immune responses.

## Experimental procedures

### Reagents, antibodies, and cell culture

Antibodies used were as follows: hamster anti-mouse CD3 (145-2C11, BioXCell), mouse anti-human CD3 (OKT3, BioXCell), mouse anti-Tyr(P) (4G10, Millipore), rabbit anti-GFP (Abcam), anti-Myc (Abcam), rabbit anti-ACK1 (Abcam, Santa Cruz Biotechnology, and Novus Biologicals), and mouse anti-HA (Cell Signaling Technology). We also used an ACK1 inhibitor (AIM-100, Calbiochem) and Indo-1 dye (Invitrogen). Lab-Tek chambered coverglasses were from Nunc. The 3× NFAT/AP1 plasmid, SLP-76-EYFP, SLP76-His, and Δ-SAM SLP-76 vectors have been described previously (22). 3Y3F-EYFP and 3Y3F-HA were subcloned into the pEYFP-N1 (Clontech) and pSR-α vectors, respectively. Single mutants (HA-Y128 and HA-Y145) were cloned into pSR-α. ACK1 plasmids were a kind gift from Dr. Scott A. Weed (West Virginia University). Jurkat and primary T cells were maintained in RPMI 1640 medium (Sigma) supplemented with antibiotics (penicillin/streptomycin) and 10% FBS at 37 °C and 5% CO_2_. HEK293T cells were maintained in DMEM (Sigma) with penicillin/streptomycin antibiotics and 10% FBS.

### Mice and T cell enrichment

DO11.10 mice were housed at Central Biological Services (Cambridge University). T cells were enriched from splenocytes and lymph nodes for CD4^+^ using a negative selection column kit (R&D Systems) or MACS® microbeads magnetic beads (Miltenyi Biotech). The purity of isolated CD4^+^ T cells was greater than 90%.

### Ratiometric calcium flux and cell motility assay

Preactivated and rested (24 h) primary CD4^+^ cells were transfected with Myc-ACK1 for 24 h and washed with cell loading medium (PBS with 1 mm calcium). Cells were loaded with 1.5 μm Indo-1 dye (Invitrogen) at 37 °C for 30 min. Excess free dye was removed by two washes with cell loading medium. The ratio of bound (405 nm) and free (485 nm) calcium was measured on a CyAn flow cytometer (Dako Cytomation) (48) upon TCR engagement by hamster anti-CD3 (2C11) plus anti-hamster cross-linking secondary antibody. The recorded data were analyzed and plotted using Summit software (Beckman Coulter).

To perform the motility assay, murine CD4^+^ T cells were activated with plate-bound anti-CD3 and CD28 (2 μg and 1 μg, respectively) for 48 h. Activated cells were then transfected with 2 μg of HA-ACK1 or empty vector control per 1 × 10^6^ cells using the Amaxa Nucleofector Kit (Lonza). Following transfection, cells were maintained in culture without anti-CD3/CD28 for an additional 24 h (*i.e.* rested). Cells were then tracked on ICAM-1-Fc (2 μg/ml)-coated plates. Alternatively, cells were treated with 2 μm of ACK1 inhibitor (AIM-100) for 30 min before imaging. Images were acquired every 10 s for 20 min. Images were processed using Zeiss LSM510 confocal software and analyzed by Volocity software (Improvision).

### Transfections, Western blotting, immunoprecipitation, luciferase promoter assay, and RT-PCR

Primary T cells were transfected by microporation using the Amaxa Nucleofector Kit (Lonza) according to the instructions of the supplier, with modifications. Prior to pulsing, the cells were incubated on ice for 10 min and for an additional minute in Amaxa solution A plus B (4.5:1) with plasmid(s) at room temperature. Jurkat cells were transfected as described previously (49) for 24 h. 24 h after transfection, the cells were stimulated with soluble anti-CD3 for 5 min. Cell lysates were then prepared on ice for immunoprecipitation. HEK293T cells were transfected using PEI by mixing with 2 μg of DNA in Opti-MEM (without FBS and antibiotics), followed by incubation at room temperature for 7 min, after which the mixture was added to cells dropwise. Cells were harvested after 24 h, and lysates were prepared for immunoprecipitation and Western blotting as described previously (49). Tyrosine phosphorylation blots were probed with anti-Tyr(P) (4G10) antibody. For the NFAT-AP1 promoter activity assay, the luciferase reporter plasmid containing 3×NFAT/AP1 binding sites was co-transfected with the control *Renilla* plasmid pRL-TK (Promega) together with ACK1 or empty vector into Jurkat cells for 24 h. 24 h after transfection, the cells were incubated with soluble anti-CD3 for an additional 6 h, and promoter activity was measured using a Dual-Luciferase assay kit (Promega) on a MicroLumat Luminator (Berthold). For real-time qPCR, RNA was isolated using the RNAeasy kit (Qiagen) and converted to cDNA using the cDNA reverse transcription kit (Applied Biosystem). Expression was quantified using commercially available primer sets against ACK1 and GAPDH (Sigma) on an AB Applied 7500 instrument according to the instructions of the manufacturer. All data are presented as relative expression levels normalized to GAPDH expression.

### In situ proximity ligation assay

PLA was performed using Duolink *in situ* PLA reagents (50) in HEK293T cells transfected with Myc-ACK1 (to be detected by anti-Myc antibody), and HA-SLP-76 (to be detected by anti-HA antibody). At 24 h, cells on slides were blocked with Duolink blocking stock, followed by the application of two PLA probes in 1× antibody diluent (anti-Myc plus anti-HA). The slides were washed for 5 min twice in wash buffer (1× TBS-T, Tris-buffered saline-Tween 20) and processed for hybridization using Duolink hybridization stock, followed by incubation for 15 min at 37 °C. Duolink ligation was performed with ligase for 15 min at 37 °C. Amplification was then achieved using Duolink amplification stock containing polymerase for 90 min at 37 °C. DNA was stained with DAPI. Each dot represents the close proximity of two interacting proteins within the cells.

### Confocal microscopy

HA-ACK1 and SLP76-EYFP were co-transfected into Jurkat T cells by electroporation (unipolar pulse, 310 V; 10 ms on a BTX electroporator). Cells were then incubated at 37 °C overnight. Prior to staining, cells were stimulated by soluble or coverslip-precoated anti-CD3 (2 μg) and fixed in 4% formaldehyde, followed by staining with Alexa Flour 647-conjugated anti-mouse Ig (Molecular Probes) to detect mouse anti-HA antibody. Cell images were acquired using a Nikon A1R confocal microscope with a plan-apochromatic violet-corrected 1.4 numerical aperture ×60 magnifying oil immersion objective. Images were acquired using one-way sequential line scans. YFP was excited at 488 nm with a laser power of 6.8 arbitrary units (AU), and its emission was collected at 525/50 nm with a PMT gain of 120 AU. The HA-ACK1 signal was excited at 637.3 nm with a laser power of 20.8 AU and collected at 595/50 nm with a PMT gain of 129 AU. The axial step size was 300 nm, with 20–40 image planes per z stack. No offset was applied on the images (51).

### Colocalization analysis

To quantify the degree of colocalization between HA-ACK1 and SLP76-EYFP, Pearson's correlation coefficient was evaluated by using the co-localization threshold plugin in ImageJ software (52) with autothreshold settings (53). 3D microscopic images were used for the analysis, where PPC values were obtained to indicate co-localization.

### Statistical analysis

All statistical analyses were performed using Prism 6 software (GraphPad Software). One-way analysis of variance and unpaired Student's *t* test were used to test the significance of changes between groups.

## Author contributions

C. E. R. conceptualized and coordinated the study and helped to write the manuscript. Y. R. T. hypothesized, designed, and performed the experiments, interpreted the results, prepared the figures, and wrote the manuscript. M. R. carried out the *in situ* PLA and biochemical binding experiment. A. R. performed the imaging experiment, analyzed the motility data, and prepared the figures. N. F. helped to conceive the study, designed the bioimaging experiments, prepared the figures, and edited the manuscript. A. J. helped with the imaging experiments. M. W. provided resources to run the confocal imaging experiment.

## Supplementary Material

Supplemental Data

## Footnotes

3 S. L. McArdel and A. H. Sharpe, unpublished data.

## Footnotes

2 The abbreviations used are:

TCR: T cell receptor
LAT: linker for the activation of T cells
SAM: sterile α motif
SH: Src homology
3Y: Tyr-113, Tyr-128, and Tyr-145
H: helix
PLA: proximity ligation assay
AU: arbitrary unit(s)
NFAT: nuclear factor of activated T cells.
