# Supplementary material for: Activated Cdc42-associated kinase 1 (ACK1) binds the sterile α motif (SAM) domain of the adaptor SLP-76 and phosphorylates proximal tyrosines
Source: J Biol Chem. 2017 Feb 10;292(15):6281–90. doi: 10.1074/jbc.M116.759555 (PMC5391757; doi:10.1074/jbc.M116.759555)
Supplement: Supplemental Data [file supp_292_15_6281__index.html]

Activated Cdc42-associated kinase 1 (ACK1) binds the SAM domain of adaptor SLP-76 and phosphorylates proximal tyrosines — Activated Cdc42-associated kinase 1 (ACK1) binds the sterile α motif (SAM) domain of the adaptor SLP-76 and phosphorylates proximal tyrosines — Ack1 regulates SLP-76 phosphorylation — Supplemental Data 

# Activated Cdc42-associated kinase 1 (ACK1) binds the sterile α motif (SAM) domain of the adaptor SLP-76 and phosphorylates proximal tyrosines

## Supplemental Data

- supplementary figure 1 (.pdf, 848 KB) - supp figure and legend
