# Supplementary material for: Activated Cdc42-associated kinase 1 (ACK1) binds the sterile α motif (SAM) domain of the adaptor SLP-76 and phosphorylates proximal tyrosines
Source: J Biol Chem. 2017 Feb 10;292(15):6281–90. doi: 10.1074/jbc.M116.759555 (PMC5391757; doi:10.1074/jbc.M116.759555)

Supplementary Fig S1. ACK1 and ACK2 expression in T-cells (A) Left panel, quantitative RT-PCR measurement of ACK1 expression in $CD4^+$ primary T-cells upon anti-CD3 stimulation in primary T-cells. Middle panel, ACK1 (*red*) and ACK2 (*silver*) expression from Immgen array expression database (CD4 T conventional (Tcon), T Regulatory (Treg) and transgenic pancreatic-infiltrating T-cells (BDC)). Right panel, RNA-seq data from expression array (http://www.th-express.org/#browse), showing fold change in RNA level as compared to naïve $CD4^+$ cells (40). (B) Western blot showing ACK1 protein expression in various cell types. (C) Sequence alignment of human and mouse ACK1 (Tnk2) proteins. (D) Antibody specificity controls for confocal microscopy experiments shown in figure 3.

Supplementary Figure S1

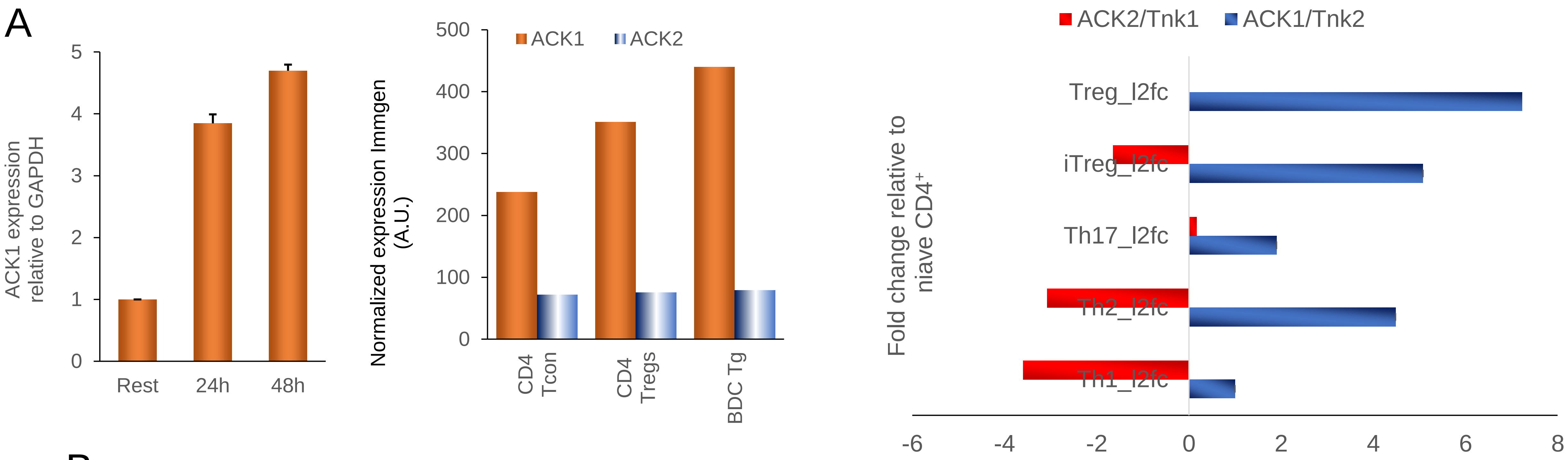

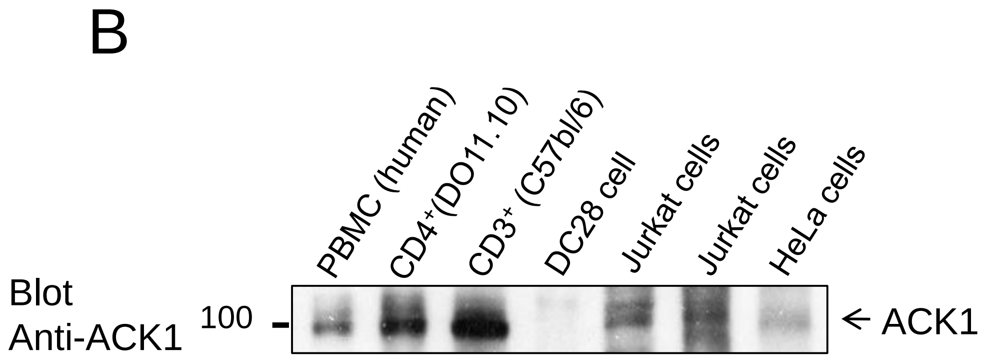

C

```
                1                                                                                                  100
ACK1_MOUSE    (1) MQPEEGTGWLLELLSEVQLQQYFLRLRDDLNITRLSHFEYVKNEDLEKIGMGRPGQRRLWEAVKRRKAMCKRKSWMSKVFSGKRLEAEFPSQHSQSTFRK
ACK1_HUMAN    (1) MQPEEGTGWLLELLSEVQLQQYFLRLRDDLNVTRLSHFEYVKNEDLEKIGMGRPGQRRLWEAVKRRKALCKRKSWMSKVFSGKRLEAEFPPHHSQSTFRK
 Consensus    (1) MQPEEGTGWLLELLSEVQLQQYFLRLRDDLNITRLSHFEYVKNEDLEKIGMGRPGQRRLWEAVKRRKALCKRKSWMSKVFSGKRLEAEFP  HSQSTFRK
                  101                                                                                                200
ACK1_MOUSE  (101) PSPTPGSLPGEGTLQSLTCLIGEKDLRLLEKLGDGSFGVVRRGEWDAPAGKTVSVAVKCLKPDVLSQPEAMDDFIREVNAMHSLDHRNLIRLYGVVLTLP
ACK1_HUMAN  (101) TSPAPGGPAGEGPLQSLTCLIGEKDLRLLEKLGDGSFGVVRRGEWDAPSGKTVSVAVKCLKPDVLSQPEAMDDFIREVNAMHSLDHRNLIRLYGVVLTPP
 Consensus  (101)  SP PG   GEG LQSLTCLIGEKDLRLLEKLGDGSFGVVRRGEWDAPAGKTVSVAVKCLKPDVLSQPEAMDDFIREVNAMHSLDHRNLIRLYGVVLT P
                  201                                                                                                300
ACK1_MOUSE  (201) MKMVTELAPLGSLLDRLRKHQGHFLLGTLSRYAVQVAEGMAYLESKRFIHRDLAARNLLLATRDLVKIGDFGLMRALPQNDDHYVMQEHRKVPFAWCAPE
ACK1_HUMAN  (201) MKMVTELAPLGSLLDRLRKHQGHFLLGTLSRYAVQVAEGMGYLESKRFIHRDLAARNLLLATRDLVKIGDFGLMRALPQNDDHYVMQEHRKVPFAWCAPE
 Consensus  (201) MKMVTELAPLGSLLDRLRKHQGHFLLGTLSRYAVQVAEGMAYLESKRFIHRDLAARNLLLATRDLVKIGDFGLMRALPQNDDHYVMQEHRKVPFAWCAPE
                  301                                                                                                400
ACK1_MOUSE  (301) SLKTRTFSHASDTWMFGVTLWEMFTYGQEPWIGLNGSQILHKIDKEGERLPRPEDCPQDIYNVMVQCWAHKPEDRPTFVALRDFLLEAQPTDMRALQDFE
ACK1_HUMAN  (301) SLKTRTFSHASDTWMFGVTLWEMFTYGQEPWIGLNGSQILHKIDKEGERLPRPEDCPQDIYNVMVQCWAHKPEDRPTFVALRDFLLEAQPTDMRALQDFE
 Consensus  (301) SLKTRTFSHASDTWMFGVTLWEMFTYGQEPWIGLNGSQILHKIDKEGERLPRPEDCPQDIYNVMVQCWAHKPEDRPTFVALRDFLLEAQPTDMRALQDFE
                  401                                                                                                500
ACK1_MOUSE  (401) EPDKLHIQMNDVITVIEGRAENYWWRGQNTRTLCVGPFPRNVVTSVAGLSAQDISQPLQNSFIHTGHGDSDPRHCWGFPDRIDELYLGNPMDPPDLLSVE
ACK1_HUMAN  (401) EPDKLHIQMNDVITVIEGRAENYWWRGQNTRTLCVGPFPRNVVTSVAGLSAQDISQPLQNSFIHTGHGDSDPRHCWGFPDRIDELYLGNPMDPPDLLSVE
 Consensus  (401) EPDKLHIQMNDVITVIEGRAENYWWRGQNTRTLCVGPFPRNVVTSVAGLSAQDISQPLQNSFIHTGHGDSDPRHCWGFPDRIDELYLGNPMDPPDLLSVE
                  501                                                                                                600
ACK1_MOUSE  (501) LSTSRPTQHLGRVKREPPPRPPQPAIFTQKTTYDPVSEDPDPLSSDFKRLGLRKPALPRGLWLAKPSARVPGTKADRSSGGEVTLIDFGEEPVVPTPRPC
ACK1_HUMAN  (501) LSTSRPPQHLGGVKKP---------------TYDPVSEDQDPLSSDFKRLGLRKPGLPRGLWLAKPSARVPGTKASRGSGAEVTLIDFGEEPVVPALRPC
 Consensus  (501) LSTSRP QHLG VKK                TYDPVSED DPLSSDFKRLGLRKPALPRGLWLAKPSARVPGTKA R SGAEVTLIDFGEEPVVP  RPC
                  601                                                                                                700
ACK1_MOUSE  (601) APSLAQLAMDACSLLDKTPPQSPTRALPRPLHPTPVVDWDARPLPPPPAYDDVAQDEDDFEVCSINSTLVGAGLPAGPSQGETNYAFVPEQAQMPPALED
ACK1_HUMAN  (586) APSLAQLAMDACSLLDETPPQSPTRALPRPLHPTPVVDWDARPLPPPPAYDDVAQDEDDFEICSINSTLVGAGVPAGPSQGQTNYAFVPEQARPPPPLED
 Consensus  (601) APSLAQLAMDACSLLD TPPQSPTRALPRPLHPTPVVDWDARPLPPPPAYDDVAQDEDDFEICSINSTLVGAGLPAGPSQG TNYAFVPEQA  PP LED
                  701                                                                                                800
ACK1_MOUSE  (701) NLFLPPQGGGKPPSSVQTAEIFQALQQECMRQLQVPTGQLTPSPTPGGDDKPQVPPRVPIPPRPTRPRVELSPAPSGEEETSRWPGPASPPRVPPREPLS
ACK1_HUMAN  (686) NLFLPPQGGGKPPSSAQTAEIFQALQQECMRQLQAPAGSPAPSPSPGGDDKPQVPPRVPIPPRPTRPHVQLSPAPPGEEETSQWPGPASPPRVPPREPLS
 Consensus  (701) NLFLPPQGGGKPPSS QTAEIFQALQQECMRQLQ P G   PSPSPGGDDKPQVPPRVPIPPRPTRP V LSPAP GEEETS WPGPASPPRVPPREPLS
                  801                                                                                                900
ACK1_MOUSE  (801) PQGSRTPSPLVPPGSSPLPHRLSSSPGKTMPTTQSFASDPKYATPQVIQAPGPRAGPCILPIVRDGRKVSSTHYYLLPERPPYLERYQRFLREAQSPEEP
ACK1_HUMAN  (786) PQGSRTPSPLVPPGSSPLPPRLSSSPGKTMPTTQSFASDPKYATPQVIQAPGPRAGPCILPIVRDGKKVSSTHYYLLPERPSYLERYQRFLREAQSPEEP
 Consensus  (801) PQGSRTPSPLVPPGSSPLP RLSSSPGKTMPTTQSFASDPKYATPQVIQAPGPRAGPCILPIVRDGKKVSSTHYYLLPERP YLERYQRFLREAQSPEEP
                  901                                                                                               1000
ACK1_MOUSE  (901) AALPVPPLLPPPSTPAPAAPTATVRPMPQAAPDPKANFSTNNSNPGARPPSLRAAARLPQRGCPGDGQEAARPADKVQMLQAMVHGVTTEECQAALQSHS
ACK1_HUMAN  (886) TPLPVPLLLPPPSTPAPAAPTATVRPMPQAALDPKANFSTNNSNPGARPPPPRATARLPQRGCPGDGPEAGRPADKIQMA--MVHGVTTEECQAALQCHG
 Consensus  (901)   LPVP LLPPPSTPAPAAPTATVRPMPQAA DPKANFSTNNSNPGARPP  RA ARLPQRGCPGDG EAARPADKIQM   MVHGVTTEECQAALQ H
                  1001                                                   1055
ACK1_MOUSE (1001) WSVQRAAQYLKVEQLFGLGLRPRVECHKVLEMFDWNLEQAGCHLLGSCGPAHHKR
ACK1_HUMAN  (984) WSVQRAAQYLKVEQLFGLGLRPRGECHKVLEMFDWNLEQAGCHLLGSWGPAHHKR
 Consensus (1001) WSVQRAAQYLKVEQLFGLGLRPR ECHKVLEMFDWNLEQAGCHLLGS GPAHHKR
```

D

Controls for antibody specificity: single transfected cells

Transfection | SLP76-YFP | ACK-HA | MERGE | MERGE + DAPI

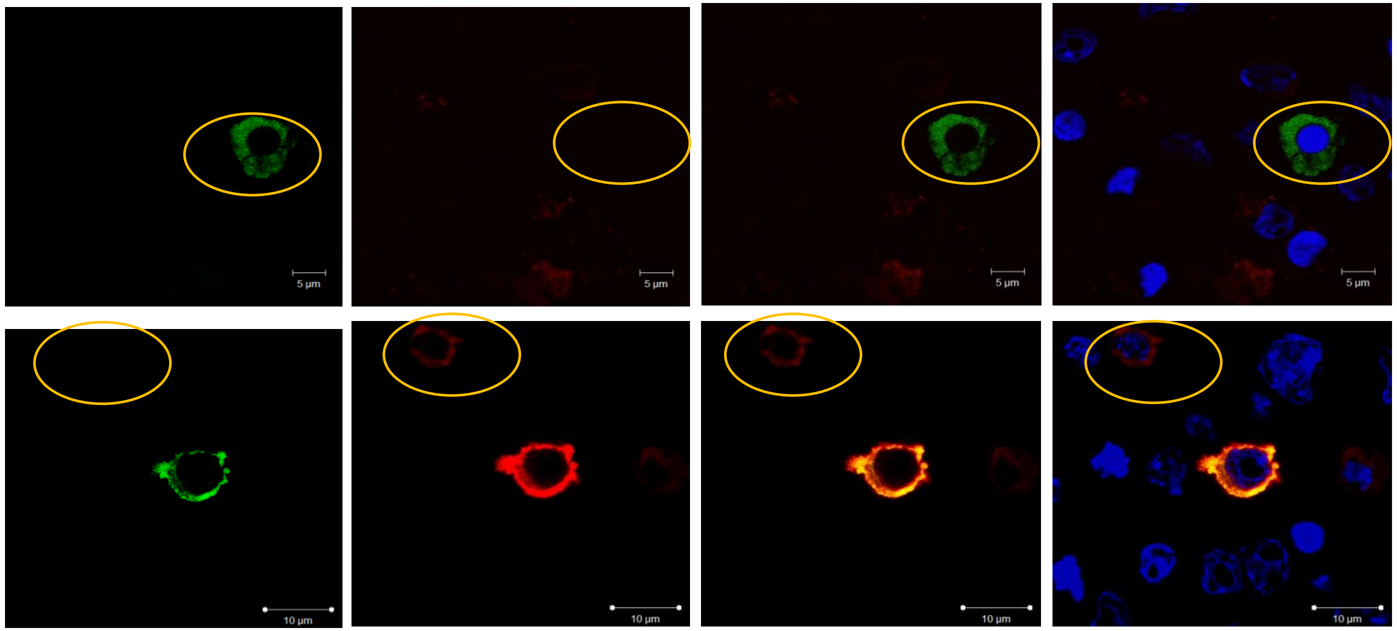

Untransfected but stimulated with mouse OKT3 antibody (5 mins) Jurkat cells

Anti-mouse 647 | MERGE | MERGE + DAPI

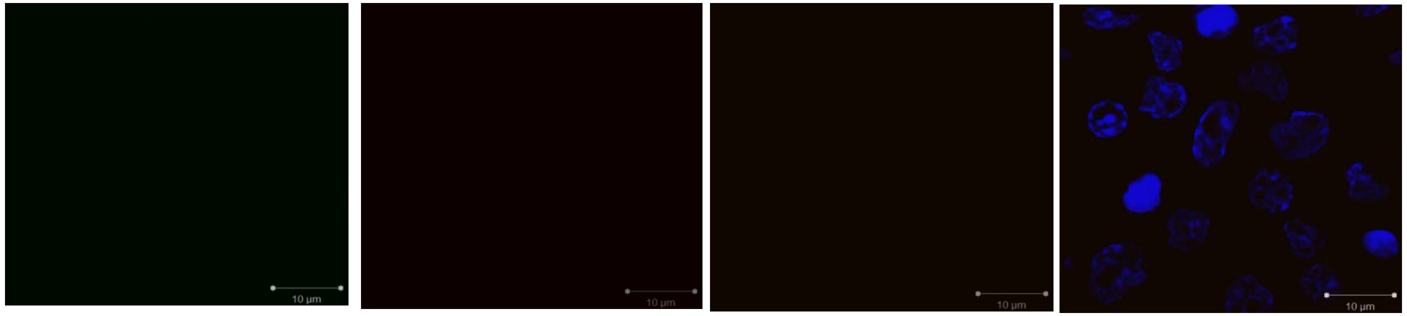

Supplement: Supplemental Data [file 10.1074_M116.759555_jbc.M116.759555-1.pdf]
